# Reduced mtDNA Copy Number Links to Vascular Calcification and Restores After Transplantation

**DOI:** 10.3390/cells14120917

**Published:** 2025-06-18

**Authors:** Angelina Schwarz, Abdul Rashid Qureshi, Leah Hernandez, Lars Wennberg, Annika Wernerson, Karolina Kublickiene, Paul G. Shiels, Roberta Filograna, Peter Stenvinkel, Anna Witasp

**Affiliations:** 1 Karolinska Institutet, Department of Clinical Science, Intervention and Technology, Division of Renal Medicine, SE-141 52 Huddinge, Swedenpeter.stenvinkel@ki.se (P.S.); anna.witasp@ki.se (A.W.); 2 Karolinska Institutet, Department of Clinical Science, Intervention and Technology, Division of Transplantation Surgery, SE-141 52 Huddinge, Sweden; 3 Glasgow Geroscience Group, School of Molecular Biosciences, University of Glasgow, Glasgow G12 8QQ, UK; 4 Karolinska Institutet, Department of Medical Biochemistry and Biophysics, SE-171 65 Solna, Sweden

**Keywords:** CKD, early vascular calcification, mtDNA copy number, renal transplantation, CAC score

## Abstract

Patients with chronic kidney disease (CKD) face an increased risk of early vascular aging, progressive vascular calcification, and premature death. With increasing age, mitochondrial function and mitochondrial DNA copy number (mtDNA-cn) decline. This has been identified as an independent predictor of frailty and mortality in cardiovascular diseases (CVDs) and cancer. However, the relationship between mtDNA-cn and vascular calcification in the context of a uremic milieu remains ambiguous. We hypothesize that a lower mtDNA-cn is associated with medial calcification, as both are linked to impaired vascular health and accelerated aging. mtDNA-cn was analyzed in 211 CKD5 patients undergoing renal transplantation (RTx) and 196 healthy controls using quantitative PCR (qPCR) for three mtDNA genes (*mtND1*, *mtND4*, and *mtCOX1*) and single-locus nuclear gene hemoglobin beta (*HbB*). In 32 patients, mtDNA-cn was also quantified one year after RTx. The association between mtDNA-cn and vascular calcification scores, circulatory cell-free (ccf) mtDNA in plasma, and the surrogate marker of biological aging (skin autofluorescence) and CVD risk was assessed. mtDNA-cn was significantly lower in CKD5 patients than in controls and correlated with biological age, vascular calcification, and CVD risk. One year after RTx there was a significant recovery of mtDNA-cn in male patients compared to baseline levels. mtDNA-cn and ccf-mtDNA were inversely correlated. This prospective study provides novel insights into the link between low mtDNA-cn and vascular aging. It demonstrates that RTx restores mtDNA levels and may improve oxidative phosphorylation capacity in CKD. Further investigation is warranted to evaluate mtDNA as a biologically relevant biomarker and a potential therapeutic target for early vascular aging in the uremic environment.

## 1. Introduction

Chronic kidney disease (CKD) is a global health burden estimated to become one of the five leading causes of death by 2050 [1]. Even in the early stages of CKD, patients are at an increased risk of cardiovascular morbidity and mortality [2]. This is linked to uremia-induced changes in vascular structure and function, such as calcification and increased peripheral resistance. With disease progression, these changes may develop into arteriosclerosis and atherosclerosis [3,4], which aggregates into an early vascular aging (EVA) phenotype [5].

Vascular calcification, marked by pathological mineral deposits in the medial vessel layers, contributes to increased stiffness and vascular resistance [4,5] and serves as the main component distinguishing EVA in CKD from non-renal cardiovascular diseases (CVDs), pre-eclampsia, or cognitive impairment [6,7]. The histopathological scoring of arterial biopsies from CKD stage 5 (CKD5, last stage of CKD and equal to kidney failure) patients has shown high-grade media calcification present in 40% of patients, which predicts the risk of cardiovascular events and mortality after renal transplantation (RTx) [8]. Coronary artery calcification (CAC), characterized by the accumulation of minerals in both the intimal and medial layers of arteries supplying the heart, is also commonly observed in CKD. CAC, commencing with small calcifications that develop into bigger lamellar depositions, decreases the arterial elasticity and compliance, appears to progress continually [9], and is an independent predictor of increased CVD risk, adverse kidney outcome, and CKD progression [10,11].

Patients with CKD show the dysregulation of multiple biological processes, such as uremic toxin retention, increased oxidative stress, vascular dysfunction, inflammation, calcification, cellular senescence, and mitochondrial dysfunction, which likely contribute to accelerated biological aging, particularly in the vasculature [5]. Although the role of mitochondria in these processes is becoming increasingly recognized, the details are still not well understood. Mitochondria are essential to cellular redox homeostasis and are thus vital for coping with fluctuations in stress and energy demand [5]. With aging, mitochondrial dysfunction ensues, and redox homeostasis is compromised, leading to increased reactive oxygen species (ROS) generation and inflammation [12], both known drivers of EVA [5]. Moreover, mitochondrial dysfunction induces a modified senescence-associated secretory phenotype (SASP), distinct from the interleukin-1 (IL1)-dependent inflammatory arm [13]. Mitochondria house their own genome, a double-stranded circular molecule known as mitochondrial DNA (mtDNA), which encodes essential components of the oxidative phosphorylation (OXPHOS) machinery and exists in multiple copies per cell [14]. As a stress response, the mitochondrion may increase its DNA content to maintain the OXPHOS capacity [15].

The amount of mtDNA-copy number (cn) in peripheral blood reflects the individual quantity of mitochondria rather than the quality of mitochondria. MtDNA-cn has been associated with aging and several diseases, including type-2 diabetes, neurodegenerative disorders, cancer, CVDs, and CKD [16,17,18,19,20,21]. Sexual dimorphism exists, as women appear to have higher mtDNA-cn than age-matched men [22,23]. Recent studies have demonstrated that increased mtDNA-cn in peripheral blood is independently associated with reduced CKD incidence [24], while decreased mtDNA-cn in CKD patients has been connected to increased all-cause mortality and infection-related deaths [25]. In IgA-nephropathy (IgAN), mtDNA-cn correlates with better renal function and milder histopathological lesions, suggesting that mtDNA-cn may reflect disease state, or that mitochondrial function is directly involved in disease progression and development [26]. Conversely, the presence and the level of mtDNA outside the cellular environment, released by apoptotic cells or damaged tissue, can indicate mitochondrial stress or tissue damage. When the integrity of the mitochondrial membrane has been compromised, mtDNA can be released into the cytoplasm, where it acts as a danger-associated molecular pattern (DAMP) triggering an innate immune response, leading to local or systemic inflammation [27,28]. This type of mtDNA can be found in the bloodstream and other body fluids as cell-free (cf-)mtDNA or circulating cell-free (ccf-)mtDNA [29].

Here, we hypothesize that decreased mtDNA-cn—as a surrogate marker of reduced mitochondrial capacity—is associated with vascular aging in the context of advanced CKD. For this purpose, we utilized a well-characterized prospective cohort of CKD 5 patients undergoing RTx [30] to study mtDNA-cn in relation to phenotypic markers of early vascular aging, surrogate markers of biological aging, and cardiovascular outcomes. Moreover, as we have previously reported that RTx decreases the biological age acceleration observed in CKD [31], we hypothesize that RTx restores mtDNA-cn back to levels comparable to those of non-uremic individuals. Thus, the quantification of mtDNA-cn was performed both prior to and one year after RTx.

## 2. Materials and Methods

### 2.1. Patients and Controls

Patients undergoing living donor (LD) RTx at Karolinska University Hospital Huddinge, Sweden, were included in an ongoing prospective study of CKD5 patients, as previously described [30]. Living kidney donors, who underwent a thorough health assessment, were included as a healthy control group. The Swedish Ethical Review Authority (EPM) approved the study protocols that were performed in accordance with the Declaration of Helsinki. Written informed consent was obtained from all participants.

**Figure 1:**
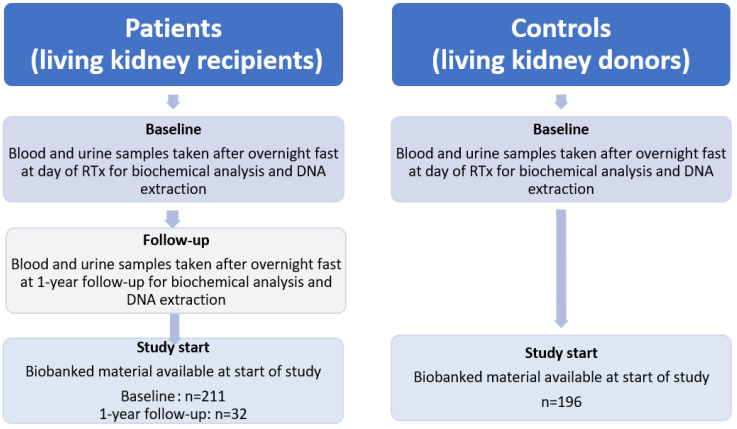
Flowchart of patient inclusion and sample availability.

For the present study, patients and controls were selected solely based on DNA availability; age and sex matching were not applied. DNA samples were available for 211 CKD5 patients and 196 controls, as well as 32 CKD5 patients at 1-year follow-up (Figure 1).

### 2.2. Biochemical Measurements

Venous blood samples after an overnight fast were taken before RTx and at one-year follow-up. The Karolinska Hospital University Laboratory Huddinge analyzed concentrations of serum creatinine, serum albumin, high-sensitivity C-reactive protein, calcium, potassium, sodium, phosphate, troponin, total cholesterol and H-cholesterol, triglyceride, high- and low-density lipoprotein (HDL-cholesterol (c), LDL-c), urea, hemoglobin, homocysteine, trimethylamine oxide (TMAO), and intact parathyroid hormone (iPTH), as previously described [11].

### 2.3. Clinical Information and Health Assessments

Age, body mass index (BMI), blood pressure, and presence of comorbidities, including diabetes mellitus (DM), CVD, and hypertension, were obtained from patients’ medical records at baseline (n = 211) and one year after RTx (n = 32). BMI was calculated as the patient’s body weight in kilograms per square of the patient’s height in meters (kg/m^2^). CVD was defined as clinical signs of cerebrovascular, cardiovascular, and/or peripheral vascular disease. Subjective global assessment (SGA) of nutritional status was evaluated using questionnaire and physical examination [32]. Handgrip strength (HGS) was determined in both hands by using a Harpenden Handgrip Dynamometer (Yamar, Jackson, MI, USA). Skin autofluorescence (SAF) was measured as a proxy of advanced glycation end-products (AGEs), using an Autofluorescence AGE reader (DiagnOptics Technologies BV, Groningen, The Netherlands) as previously described [31,33].

### 2.4. Cardiovascular Assessments

To assess the coronary artery calcification (CAC) score, patients underwent non-contrast multi-detector cardiac CT scanning (LightSpeed VCT or Revolution CT; GE Healthcare, Milwaukee, WI, USA) with standard ECG-gated protocol, using semi-automatic software (syngo CT VC28) one week prior to RTx (syngo.via CT Ca Scoring, Siemens Healthcare, Erlangen, Germany). CAC score was assessed as a lesion with an area >1 mm^2^ and peak intensity > 130 Hounsfield units (HU) based on the Agatston method and expressed in Agatston units (AU) [34].

Aortic augmentation index corrected for heart rate was calculated from multiple non-invasive SphygmoCor measurements performed one week prior to RTx [33].

Framingham cardiovascular disease risk score (FRS), an estimate of 10-year risk of developing CVD, was calculated from chronological age and sex stratified tables with scores for diabetes, systolic blood pressure (SBP), anti-hypertensive medication, total cholesterol, HDL cholesterol, and smoking habit [35].

Medial calcification was determined by an experienced pathologist on epigastric arteries biopsied during the RTx. Arteries were formalin-fixed, paraffin-embedded, and stained with hematoxylin and eosin and von Kossa staining. The degree of medial calcification was semiquantified on the von Kossa-stained sections and graded 0 to 3, where 0 indicated no calcification and 3 indicated extensive medial calcification [30].

### 2.5. Quantitative PCR (qPCR) with TaqMan^®^ Probes

Whole blood was collected at study inclusion from both patients and controls as well as one year after transplantation from patients. DNA extraction was performed at the Karolinska Institutet Biobank Core Facility, using a high-throughput automated protocol with Chemagen^®^ magnet bead extraction kit. The Taqman probes (ThermoFisher, Waltham, MA, USA) were tested on a DNA dilution series to determine the appropriate DNA input, allotted to 5 ng per reaction. qPCR was performed on a QuantStudio^TM^ 7 Flex Real-Time PCR system with the QuantStudio^TM^ Real-Time PCR Software version 1.3. Three different TaqMan probes targeting the mtDNA-encoded genes, NADH: ubiquinone oxidoreductase core subunit 1 (Complex1) (*mtND1*, #Hs02596873_s1), NADH: ubiquinone oxidoreductase core subunit 4 (Complex1) (*mtND4*, #Hs02596876_g1), and cytochrome c oxidase I (*mtCOX1*, #Hs02596864_g1), were used. Two TaqMan probes for nuclear DNA single-locus encoded genes were also used: *18S rRNA* (#Hs99999901_s1) and hemoglobin beta (*HbB*, #Hs00758889_s1). Reactions were run in triplicate. To calculate the relative cn of mtDNA, the delta-delta CT method was used, with *18S rRNA* as reference gene to subtract the CT values, and *HbB* was used for normalization [36].

### 2.6. Measurement of ccf-DNA

Circulating ccf-DNA was quantified using plasma samples from CKD5 patients within the LD-RTx patient cohort. DNA was isolated from plasma using QIAamp DNA Mini Kit (Qiagen, Hilden, Germany) according to the manufacturer’s protocol. Total cfDNA was quantified using fluorometric, high-sensitivity dsDNA kit Qubit Fluorometer and Qubit dsDNA HS Assay Kit (Invitrogen, Carlsbad, CA, USA) according to manufacturer’s protocol. Subcellular origin of cfDNA in isolates was determined by real-time PCR on QuantStudio 7 Flex Real-Time PCR Systems (Applied Biosystems, Waltham, MA, USA) using the SsoAdvanced Universal SYBR Green Supermix (Bio-Rad, Hercules, CA, USA). Primers encoding human beta-globin gene and primers targeting D-loop amplification were used to quantify nuclear-cfDNA and mt-cfDNA, respectively, as previously described [37].

### 2.7. Statistical Analysis

Normality of all variables was assessed with Shapiro–Wilk test. Variables are presented as percentages or medians with corresponding interquartile ranges. Statistical significance was set at *p* < 0.05. *p*-values were not adjusted for multiple comparisons and are presented descriptively. For continuous variables, group comparisons included the non-parametric Mann–Whitney U-test (comparison between two groups) or Kruskal–Wallis test (comparison between more than two groups). The Wilcoxon signed-rank test was utilized to test differences between paired samples. For nominal variables, Fischer’s exact test or chi-square was used. Univariate analyses were performed using the non-parametric Spearman rank correlation test. Predictors of medical vascular calcification were assessed in a multivariate logistic regression model including sex, age, and *mtND4* tertiles. The individual *mtND4*-cn relations with clinical parameters were visualized through a Sankey plot. As we have not performed multiple testing correction, it may increase the risk of type I error. All statistical analyses were conducted using Stata 18.0 (Stata Corporation, College Station, TX, USA) and SAS version 9.4 service 7 (SAS Campus Drive, Cary, NC, USA).

## 3. Results

### 3.1. Patient Disposition

Descriptive data for patients and controls, stratified by sex, are presented in Table 1. The sex distribution differed between patients and controls, with 67% (n = 142) male CKD patients compared to 38% (n = 73) male controls (*p* < 0.0001). In the patient group, the reported underlying causes of CKD were diabetic nephropathy (n = 11) 5%, glomerulonephritis (n = 86) 40%, adult polycystic kidney disease (n = 34) 16%, and malignant hypertension or renal vascular disease (n = 28) 13%, whereas 25% (n = 52) had CKD due to an unknown cause or other reasons. Twenty patients (10%) had diabetes mellitus. Out of the 211 patients, 82 patients (39%) received their transplant preemptively, i.e., without dialysis, while 62 patients (29%) had received hemodialysis, and 56 patients (27%) had peritoneal dialysis prior to RTx.

### 3.2. mtDNA Genes Show Similar Distribution

**Figure 2:**
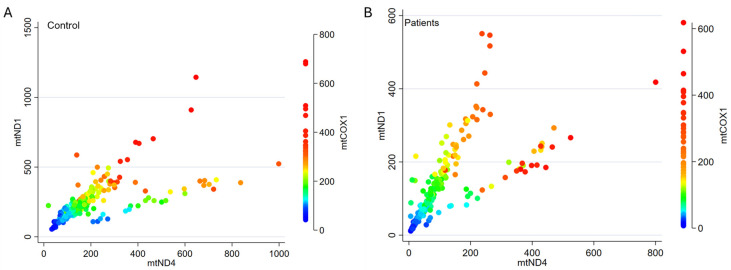
Correlation of cn values between the three different mitochondrial genes *mtND1*, *mtND4*, and *mtCOX1* for each control (**A**) and each CKD5 patient (**B**). The *p*-values for all individual correlations (Spearman’s rho) within each group are <0.0001.

To check the validity of the selected TaqMan assays, we correlated the cn values for all three mitochondrial genes, *mtND1*, *mtND4*, and *mtCOX1*, in CKD5 patients and RTx donors. Clear correlations between the genes were observed in both groups (Figure 2).

### 3.3. mtDNA-cn Is Significantly Lower in CKD5 Patients than in Controls

**Figure 3:**
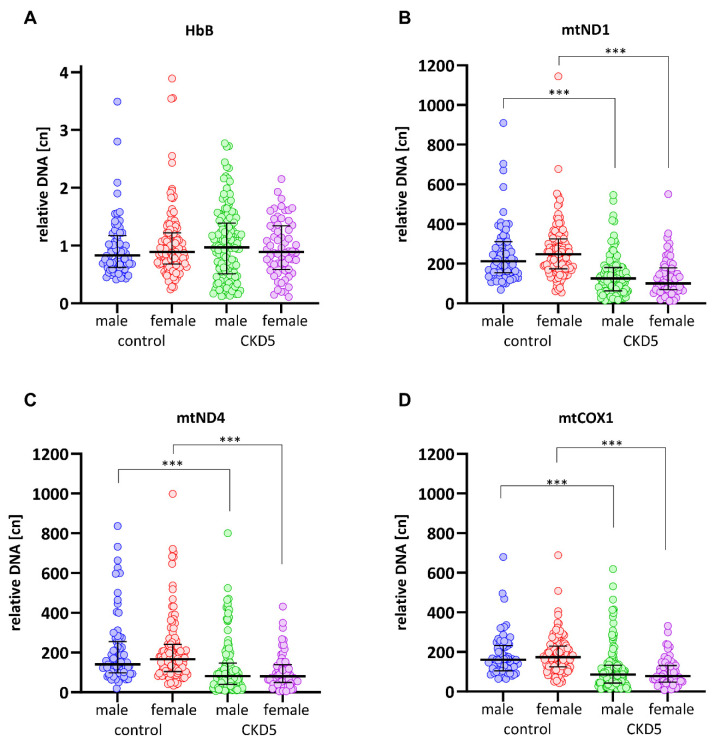
The mtDNA-cn values are significantly lower in CKD5 patients than in controls, while the cn values for *HbB* (**A**) are similar between groups. mtDNA-cn values according to *mtND1* (**B**), *mtND4* (**C**), and *mtCOX1* (**D**) do not significantly differ between males and females. Results are presented as medians with an interquartile range, and *p*-values are obtained from a Kruskal–Wallis test with Dunn’s multiple comparison. *** = *p* < 0.0001.

Overall, CKD5 patients had significantly lower mtDNA-cn values compared to the controls, while the cn for single-locus nuclear gene *HbB* did not differ (Table 2). Disease etiology was not associated with mtDNA-cn (Supplemental Figure S1). Both male and female patients presented with significantly lower mtDNA-cn values for all three investigated mt-genes compared to the controls. In both groups, no significant difference in cn values was seen according to sex (Figure 3).

Within the patient group, there were no significant cn differences between patients with (n = 20) or without (n = 181; missing n = 10) diabetes: *mtND1* 121 vs. 126, *p* = 0.981; *mtND4* 73 vs. 86, *p* = 0.359; and *mtCOX1* 76 vs. 92, *p* = 0.166 (Supplemental Figure S2).

### 3.4. mtDNA-cn Is Inversely Correlated with ccf-mtDNA

**Figure 4:**
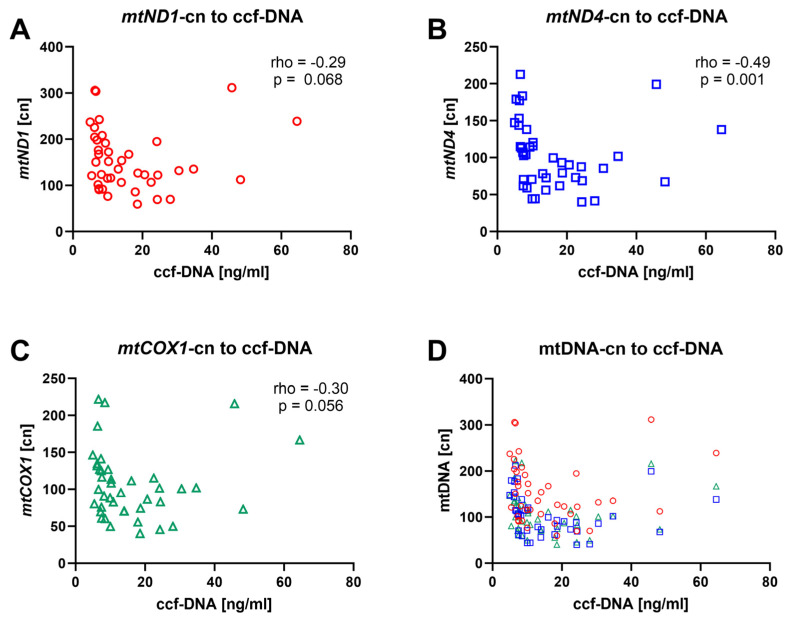
Spearman correlation between mitochondrial ccf-DNA in serum and mtDNA-cn in whole blood from 41 CKD5 patients. mtDNA-cn according to (**A**) *mtND1* (red), (**B**) *mtND4* (blue), (**C**) *mtCOX1* (green), and (**D**) all correlations overlayed in one graph.

Next, we investigated the association between mtDNA-cn and ccf-mtDNA measured in serum from a subgroup of 41 patients [37]. Patients with lower ccf-mtDNA tended to have higher cn values of all three investigated mtDNA genes (Figure 4). While the correlations between ccf-mtDNA and *mtND1*-cn and *mtCOX1*-cn were non-significant or borderline significant, a significant inverse correlation was observed between *mtND4*-cn and ccf-mtDNA (rho = −0.49, *p* = 0.001). This inverse correlation suggests that ccf-mtDNA levels in plasma represent a marker of cellular damage, inflammation, or mitochondrial health.

### 3.5. CAC Score, Medial Calcification, and Biological Age by SAF Correlate to mtDNA-cn

Given that a high mtDNA-cn has been linked to good health and longevity, we analyzed mtDNA-cn values in relation to various biological measures linked to pathological aging, including medial calcification score, CAC score, and biological age determined through the SAF method. Table 3 presents the distribution of age-associated variables across *mtND4*-cn tertiles. Significant correlations were observed between *mtND4*-cn’s and media calcification and CAC scores as well as biological age as assessed by SAF. In addition, *mtND4*-cn was associated with the FRS. Similar correlations were also noted for *mtND1* and *mtCOX1*, although they were less pronounced (see Supplemental Tables S1 and S2). When we performed a multivariate regression analysis with media calcification as the dependent variable, we found that only age and sex were significantly associated (Supplemental Table S3).

### 3.6. Significant Recovery of mtDNA-cn After RTx

**Figure 5:**
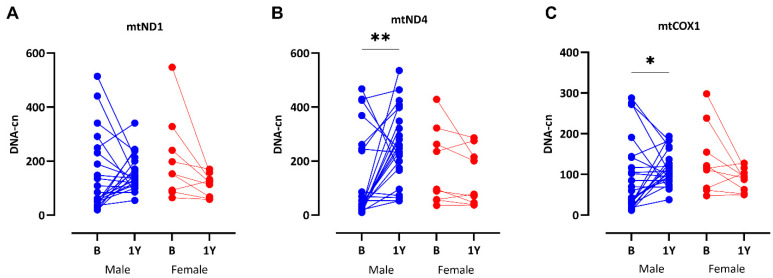
Significant recovery of mtDNA-cn levels in males one year after RTx: *mtND1* (**A**), *mtND4* (**B**), and *mtCOX1* (**C**). Differences between time-points (B = basal, 1Y = one year after transplantation) are assessed with Wilcoxon paired test. * = *p* < 0.01, ** = *p* < 0.001.

As recent data suggest that RTx may ameliorate the accelerated aging processes in CKD [31], we wanted to measure mtDNA-cn in the same patients one year after the RTx. At one year follow-up, DNA was available in 32 patients (clinical characteristics listed in Supplemental Table S3). All patients were on standard immunosuppressive treatment, i.e., prednisolone, mycophenolate, and tacrolimus. While a significant increase in both *mtND4* (*p* = 0.002) and *mtCOX1* (*p* = 0.05) was observed in males, female patients’ (n = 9) mtDNA-cn values appeared to decrease, although these changes were not significant (Figure 5).

### 3.7. Patients with CVD Event Have Low mtND4-cn Values

**Figure 6:**
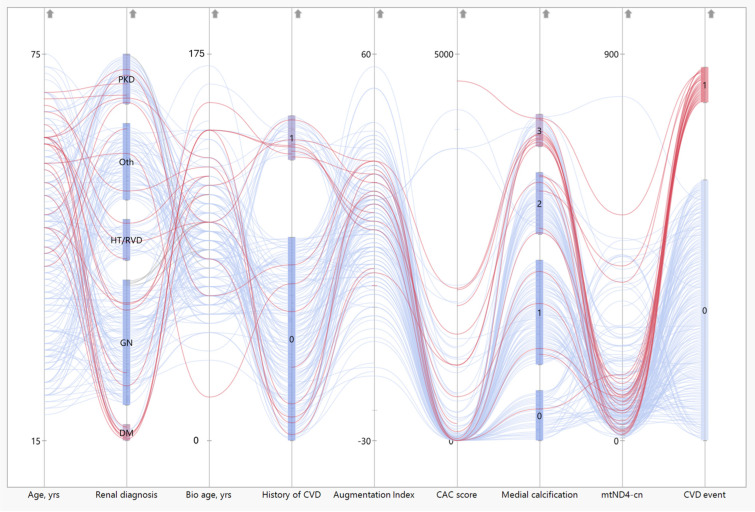
The distribution of various prognostic EVA measures and *mtND4*-cn and the occurrence of a cardiovascular disease (CVD) event after RTx. The red lines represent the patients who had an occurrence of a CVD event, while the blue lines represent the patients who had, so far, no recorded CVD event after RTx. CVD events are defined as a composite endpoint encompassing serious heart-related conditions, including nonfatal myocardial infarction, angina pectoris, peripheral vascular events, and cardiac failure. Yrs = years; bio age = biological age by skin autofluorescence measurement; augmentation index = aortic augmentation pressure; and CAC score = coronary artery calcification score.

To investigate the association between mtDNA-cn at baseline and the likelihood of a CVD event during follow-up, a Sankey plot was generated, incorporating different prognostic parameters for longevity and *mtND4*-cn (Figure 6). The Sankey plot illustrates the distribution, correlation, and flow of several markers directed towards one outcome, here any registered CVD event during a follow-up of 5 years. While the red color represents the flow of individual patients who had a CVD event during a 5-year follow-up, the thickness of the nodes represents the proportionality to the magnitude of the flow. CVD events were defined as a composite endpoint encompassing serious heart-related conditions, including nonfatal myocardial infarction, angina pectoris, peripheral vascular events, and cardiac failure. *mtND4*-cn was chosen as it showed the strongest correlations out of the three examined genes. While chronological age, biological age (assessed by SAF), and aortic augmentation pressure (AP) were not associated with CVD events, patients with coronary artery calcification (CAC), preexisting CVD, previous diabetic nephropathy, or low *mtND4*-cn were more likely to experience a CVD event after RTx (Figure 6).

## 4. Discussion

The significance of mitochondrial dysfunction in aging and CKD has garnered increased attention, with mtDNA-cn proposed as a key marker of mitochondrial health and OXPHOS capacity. This study assessed mtDNA-cn levels in CKD5 patients before and one year after LD-RTx. Our findings indicate that CKD5 patients had significantly lower mtDNA-cn compared to controls with partial restoration one year post-transplantation. We also observed an inverse correlation between *mtND4*-cn and ccf-DNA in 41 CKD5 patients and low mtDNA levels at baseline associated with biological age and vascular calcification. Moreover, low mtDNA levels increased the risk of a CVD event during a five-year follow-up period. Overall, this further strengthens the importance of mitochondrial capacity in the development of EVA in the uremic milieu and is in line with other observations indicating that CKD is a disease of accelerated biological aging.

Our findings support the emerging concept that mtDNA-cn may serve as a marker of biological aging and vascular health in CKD, with implications for future cardiovascular events. These results align with recent studies showing that mtDNA-cn declines with advancing CKD stages [39] and that decreased mtDNA-cn correlates with increased risk for all-cause mortality in CKD patients or higher CKD incidence [24,25]. Mitochondrial integrity is of importance for CVD pathogenesis [40,41], and mitochondrial malfunction or damage through oxidative stress is a major driver of systemic inflammation [42]. When damaged mtDNA is released into the cytosol and recognized by toll-like-receptor-9 (TLR9) as a DAMP, the NLRP3 inflammasome is triggered [43]. The NLRP3 inflammasome is linked to vascular inflammation, calcification, and cellular senescence, as observed in CVD and CKD [44,45]. We have shown that a lower amount of mtDNA-cn correlated with a worsened vascular risk profile, such as media calcification, CAC score, and the FRS. Thus, measuring mtDNA—whether as ccf-DNA in plasma or mtDNA-cn in whole blood—offers valuable insight into an individual’s vascular health and CVD risk. An even greater promise would be the combination of measuring both variants of mtDNA to create a surrogate marker of biological age, CVD risk, and inflammation state.

We found no association between mtDNA-cn and C-reactive protein. This may be attributed to our LD-RTx patients representing a healthier uremic group, characterized by only subtle changes in inflammation. Additionally, assessments of other proinflammatory markers, including SASP markers and microbial metabolites, may better capture aspects of the proinflammatory milieu linked to EVA in CKD patients. Notably, given that mitochondrial dysfunction is a cellular hallmark of aging, our observation is congruent with previous observations indicating that cellular markers of aging capture <10% of the inflammatory burden in a general population cohort, whereas proinflammatory microbial metabolites, such as trimethylamine, can capture a much larger percentage [46].

This study is the first to demonstrate a significant increase in mtDNA-cn in patients following RTx. Nevertheless, in the female patients it appears that some cn values even decrease below baseline values after RTx. Alas, the limited number (only nine) of female patients investigated at the one-year follow-up renders it challenging to draw definitive conclusions, and further studies are warranted.

Regardless, mtDNA-cn is emerging as a systemic index of mitochondrial biogenesis and has been reported to be sex-dependent, most likely due to a different sex hormone environment and hormonal receptor stimulation [47]. We recently reported that RTx recipients exhibited a deceleration in biological age two years post-transplant, in contrast to patients who remained on dialysis [31]. These observations are congruent with mtDNA microchimerism differences observed between RTx donors and recipients. Indeed, El-Ansary et al. [48] could show, through measuring mtDNA microchimerisms in the peripheral blood of recipients within a month after RTx, that a higher amount of donor mtDNA correlates with better immediate kidney allograft function as well as at one- and three-year follow-up time-points.

One hypothesis for the increased mtDNA-cn observed at the one-year follow-up is that it may partly reflect donor-derived mtDNA. This could help explain the absence of a similar replenishing effect in female recipients. Given that kidneys are often donated by spouses, and men averagely have lower mtDNA-cn values, this might attenuate the recovery of mtDNA-cn in female recipients. Because mtDNA-cn also shows tissue-specific sex variation, and we did not analyze the mitochondrial quantities within the kidneys, this issue remains speculative and has yet to be resolved.

The relationship between ccf-DNA and mtDNA-cn remains poorly studied [49]. Previous research on mtDNA has focused on ccf-DNA in donors and its relationship with renal allograft survival, with measurements typically conducted within one month after RTx. In CKD, increased ROS production, senescence and inflammaging are linked to endothelial dysfunction and increased arterial stiffening with the accumulation of calcifications within the vascular tree [50]. Therefore, it has been suggested that ccf-DNA reflects the inflammatory potential of mitochondrial remnants released during apoptosis or tissue damage, whereas mtDNA-cn represents the overall mitochondrial quantity in an individual, rather than the quality of the mtDNA itself [49].

In another study we measured ccf-DNA in the plasma of recipients before and after RTx focusing on differences in the pre-transplant renal replacement therapies and their influence on chronic inflammation [37]. In this study, the combined analysis of mtDNA measures revealed an inverse relationship between *mtND4*-cn and ccf-DNA, suggesting that higher mtDNA-cn and lower ccf-DNA are indicative of better health, while the opposite pattern reflects poorer health. Thus, the combination of mtDNA-cn and ccf-DNA could provide a more comprehensive and novel assessment of allograft survival probability and vascular health.

The interpretation of the presented results should be considered in view of some limitations of the study design, including the selection bias inherited in the inclusion of CKD5 patients eligible for LD-RTx and the relatively small sample size. This group of patients is in a comparatively good physical state and younger than the typical dialysis patient, making it difficult to extrapolate the results to the broader CKD5 population. Still, many recipients have undergone prolonged dialysis, which may have affected their blood mtDNA levels. Nevertheless, we found no such correlation in our patient cohort. Due to the small sample size and thus limited statistical power, we refrained from multiple testing adjustments. As we are aware that this increases the risk of type I error, we intend to confirm our results in an independent cohort. Furthermore, the groups were not age and sex matched. The uneven sex distribution among recipients and donors—where two thirds of the patients are male, while only about one third of the control group are male—and previous studies that highlight sex as a factor influencing mtDNA-cn warrants careful consideration of this discrepancy [22,23]. We stratified mtDNA-cn data by sex and found no differences between males and females at baseline. One possible explanation is that the metabolic advantages typically associated with females diminish in the toxic uremic milieu. In addition to age and sex, type-2 diabetes and metabolic syndrome have been shown to adversely impact mtDNA-cn [16,51]. In our study, only twenty patients had diabetes, and we found no correlation with mtDNA-cn. Finally, the number of patients with DNA samples available at follow-up after RTx was limited; thus, findings on mtDNA-cn values at one-year follow-up should be validated in a larger cohort.

## 5. Conclusions

In conclusion, we provide new evidence linking low mtDNA-cn to vascular aging and show that RTx restores mtDNA levels. Our findings highlight the potential of the combination of mtDNA-cn and ccf-DNA as a biologically relevant biomarker and a promising therapeutic target for early vascular aging in CKD. Furthermore, given its non-invasive nature, mtDNA measurement offers a practical and valuable tool for risk assessment, prognosis, and guiding therapeutic strategies to reduce the high cardiovascular morbidity and mortality associated with CKD.

## Figures and Tables

**Table 1:** Basic clinical parameters of the CKD5 patients and controls stratified by sex.

	All Patients	Female Patients	Male Patients	*p*-Value	N Data	All Controls	Female Controls	Male Controls	*p*-Value	N
	N = 211	N = 69	N = 142			N = 196	N = 117	N = 71		Data
Age (years)	47 (32–58)	49 (30–61)	47 (33–55)	0.52	211	51 (42–59)	51 (43–61)	48 (39–56)	<0.05	186
Bio. age by SAF (years)	90 (70–107)	97 (78–111)	86 (70–103)	0.072	174	n.a.	n.a.	n.a.	n.a.	
BMI, kg/m^2^	24.2 (22.2–26.7)	24.4 (22.5–26.5)	23.5 (21.6–27.1)	0.34	200	24.9 (23.1–27.2)	24.4 (22.5–27.2)	25.2 (23.9–27.2)	0.16	161
Systolic BP, mmHg	141 (130–155)	142 (131–156)	138 (126–154)	0.15	200	124 (117–131)	123 (116–131)	125 (117–132)	0.39	161
Diastolic BP, mmHg	85 (76–94)	83 (76–90)	86 (76–95)	0.31	200	75 (70–80)	74 (68–80)	79 (71–83)	0.014	161
Creatinine, µmol/L	720 (581–877)	598 (490–722)	775 (622–943)	<0.001	211	69 (62–80)	65 (58–70)	81 (74–87)	<0.001	160
eGFR, mL/min	7 (5–9)	7 (6–9)	7 (5–8)	0.25	202	100 (90–110)	98 (89–110)	103 (92–110)	0.22	161
Triglycerides, mmol/L	1.3 (1.0–1.9)	1.4 (1.0–2.0)	1.3 (1.0–1.9)	0.84	210	0.8 (0.7–1.3)	0.7 (0.6–1.0)	1.2 (0.8–1.6)	<0.001	146
Cholesterol, mmol/L	4.4 (3.6–5.1)	4.8 (3.9–5.6)	4.2 (3.5–4.9)	<0.001	210	5.2 (4.6–5.8)	5.2 (4.5–5.8)	5.2 (4.6–5.7)	0.94	150
HDL-cholesterol, mmol/L	1.3 (1.1–1.6)	1.6 (1.3–1.9)	1.2 (1.0–1.5)	<0.001	210	n.a.	n.a.	n.a.	n.a.	
S-Albumin g/L	34 (32–37)	33 (32–36)	35 (32–38)	0.081	209	n.a.	n.a.	n.a.	n.a.	
hsCRP, mg/L	0.9 (0.3–2.0)	0.8 (0.3–2.2)	0.9 (0.4–1.9)	0.73	209	n.a.	n.a.	n.a.	n.a.	
TMAO, μM	51 (34–89)	49 (32–94)	54 (36–88)	0.65	183	n.a.	n.a.	n.a.	n.a.	
iPTH, ng/L	260 (170–400)	240 (160–420)	270 (175–394)	0.55	209	n.a.	n.a.	n.a.	n.a.	
Calcium, mmol/L	2.3 (2.2–2.4)	2.3 (2.2–2.4)	2.3 (2.1–2.4)	0.61	209	n.a.	n.a.	n.a.	n.a.	
Phosphate, mmol/L	1.7 (1.4–2.0)	1.6 (1.4–1.9)	1.7 (1.3–2.1)	0.47	209	n.a.	n.a.	n.a.	n.a.	
Troponin-T, ng/L	22.5 (14–38)	20 (11–30)	25 (16–44)	0.006	202	n.a.	n.a.	n.a.	n.a.	
Diabetes mellitus, n (%)	20 (10.0)	5 (8.2)	15 (10.7)	0.58	201	n.a.	n.a.	n.a.	n.a.	
CAC > 0, n (%)	77 (49.7)	23 (48.9)	54 (50.0)	0.90	155	n.a.	n.a.	n.a.	n.a.	
CT-total	0 (0–126)	0 (0–137)	2 (0–97)	0.84	159	n.a.	n.a.	n.a.	n.a.	
PEW (SGA > 1), n (%)	66 (32.7)	24 (37.5)	42 (30.4)	0.32	202	n.a.	n.a.	n.a.	n.a.	
AORTIC AIX@HR75 (%)	20.2 (10.7–27.3)	23.1 (13.6–28.4)	19 (7.9–26)	0.053	127	n.a.	n.a.	n.a.	n.a.	
FRS (%)	6.9 (3.2–14.8)	4.8 (2.0–8.9)	8.0 (3.7–16.5)	0.001	201	n.a.	n.a.	n.a.	n.a.	
Hand grip strength, %	95 (78–109)	93 (74–104)	98 (81–111)	0.13	157	n.a.	n.a.	n.a.	n.a.	

Bio. age by SAF = biological age by skin autofluorescence method; BMI = body mass index; eGFR = estimated glomerular filtration rate (CKD-EPI 2021 formula [38]); BP = blood pressure; HDL = high density lipid; hsCRP = high sensitivity C-reactive protein; TMAO = trimethylamine oxide; iPTH = intact parathyroid hormone; PEW = protein-energy wasting; SGA = subjective global assessment of nutritional status; CAC = coronary artery calcium; CT total = CAC scores obtained by cardiac computed tomography [11]; aortic AIX@HR75 = aortic augmentation index corrected for heart rate; FRS = Framingham CVD risk score; n.a. = not available; and *p*-value obtained by Mann–Whitney U test.

**Table 2:** Comparison of mtDNA-cn values between CKD5 patients and controls.

	Patients (N = 211)	Controls (N = 196)	*p*-Value
*mtND1*, cn	119 (65–180)	222 (162–316)	<0.0001
*mtND4*, cn	81 (40–143)	153 (103–242)	<0.0001
*mtCOX1*, cn	85 (45–132)	166 (116–232)	<0.0001
*HbB*, cn	1.0 (0.6–1.4)	0.9 (0.7–1.2)	0.687

cn = copy number; *p*-values obtained by Mann–Whitney U test.

**Table 3:** Estimated biological age by skin autofluorescence (SAF) measurements and coronary artery calcium (CAC) score in 211 CKD5 patients according to tertiles of *mtND4*-cn.

*mtND4*cn-Tertiles	Low	Medium	High	All	N	*p*-Value
	N = 69	N = 70	N = 72	N = 211		
*mtND4*, cn	30 (18–40)	79 (66–92)	193 (135–337)	81 (40–143)	211	<0.001
Males, n (%)	43 (62.3)	50 (71.4)	49 (68.1)	142 (67.3)	211	0.51
Age (years)	51 (35–60)	49 (37–61)	41 (30–49)	47 (32–58)	211	0.012
Bio. age by SAF (years)	99 (78–115)	99 (78–111)	82 (65–95)	90 (70–107)	174	<0.001
BMI, kg/m^2^	25.1 (22.6–27.8)	24.3 (22–27.5)	23.5 (22.2–26)	24.2 (22.3–26.9)	209	0.056
Creatinine, µmol/L	686 (560–829)	684 (584–859)	736 (598–942)	720 (581–877)	211	0.39
CT-total	6 (0–361)	13 (0–282)	0 (0–14)	0 (0–126)	159	0.008
Media calcification score, n (%)					177	<0.001
0	5 (7.2)	11 (15.7)	24 (33.3)	40 (19)		
1	24 (34.8)	25 (35.7)	24 (33.3)	73 (34.6)		
2	11 (15.9)	16 (22.9)	15 (20.8)	42 (20.8)		
3	8 (11.6)	8 (11.4)	6 (8.3)	22 (10.4)		
FRS (%)	9.1 (4.2–17.2)	7.8 (4.3–19.0)	4.6 (2.3–9.1)	6.9 (3.2–14.8)	201	0.011

cn = copy number; BMI = body mass index; Bio. age by SAF = biological age assessed by skin autofluorescence; CT total = CAC (coronary artery calcium) scores obtained by cardiac computed tomography [11]; FRS = Framingham CVD risk score; and *p*-values are obtained from Kruskal–Wallis comparison.

## Data Availability

The data that support the findings of this study are not publicly available due to privacy/ethical reasons. The data will be available upon request from the corresponding author.

## Supplementary Materials

The following supporting information can be downloaded at https://www.mdpi.com/article/10.3390/cells14120917/s1, Table S1: Estimated biological age by skin autofluorescence (SAF) measurements and coronary artery calcium (CAC) score in 211 CKD5 patients according to tertiles of mtND1-cn. Table S2: Estimated biological age by skin autofluorescence (SAF) measurements and coronary artery calcium (CAC) score in 211 CKD5 patients according to tertiles of mtCOX1-cn. Table S3: Predictors of vascular medial calcification in 177 CKD5 patients. Table S4: Clinical characteristics of patients one year after kidney transplantation. Figure S1: MtDNA-cn in patients stratified according to CKD etiology. There is no significant difference in median mtDNA-cn between groups. DM = diabetes mellitus/diabetic nephropathy; GN = glomerulonephritis; HT/RVD = hypertension/renal vascular disease; oth = other causes or unknown; and ADPKD = adult polycystic kidney disease. Figure S2: MtDNA-cn in CKD5 patients stratified according to presence of diabetes mellitus (DM). There are no significant cn differences between patients with (n = 20) or without (n = 181; missing n = 10) diabetes. (A) mtND1 121 vs. 126, *p* = 0.981; (B) mtND4 73 vs. 86, *p* = 0.359; and (C) mtCOX1 76 vs. 92, *p* = 0.166. Groups are compared with Mann–Whitney U-test. Figure S3: MtDNA-cn at one-year-follow-up according to disease etiology. There is no significant difference in median mtDNA-cn at one-year-follow-up, when analyzed according to their original disease etiology before RTx. DM = diabetes mellitus/diabetic nephropathy, GN = all glomerulonephritis, HT/RVD = hypertension/renal vascular disease, Oth = other causes like genetic or unknown, ADPKD = adult polycystic kidney disease, 1år = one year.

## Abbreviations

The following abbreviations are used in this manuscript:

AP	Augmentation pressure
BMI	Body mass index
CAC	Coronary artery calcification
Ccf-	Circulatory cell-free
CKD	Chronic kidney disease
CVD	Cardiovascular disease
DAMP	Damage-associated molecular pattern
DM	Diabetes mellitus
eGFR	Estimated glomerular filtration rate
EVA	Early vascular aging
FRS	Framingham cardiovascular disease risk score
HbB	Hemoglobin beta
HDL-c	High-density lipoprotein cholesterol
HGS	Handgrip strength
IgAN	IgA Nephropathy
LD	Living donor
LDL-c	Low-density lipoprotein cholesterol
mtDNA-cn	Mitochondrial DNA copy number
OXPHOS	Oxidative phosphorylation
PEW	Protein-energy wasting
ROS	Reactive oxygen species
RTx	Renal transplantation
SAF	Skin autofluorescence
SASP	Senescence-associated secretory phenotype
SBP	Systolic blood pressure
SGA	Subjective global assessment of nutritional status
