# Supplementary material for: Reduced mtDNA Copy Number Links to Vascular Calcification and Restores After Transplantation
Source: Cells. 2025 Jun 18;14(12):917. doi: 10.3390/cells14120917 (PMC12190628; doi:10.3390/cells14120917)
Supplement: Supplementary file 1 [file cells-14-00917-s001.zip › cells-3674036-supplementary.pdf]

cells

MDPI

Article

Reduced mtDNA Copy Number Links to Vascular Calcification and Restores After Transplantation

Angelina Schwarz[1,*], Abdul Rashid Qureshi[1], Leah Hernandez[1], Lars Wennberg[2], Annika Wernerson[1], Karolina Kublickiene[1], Paul G. Shiels[3], Roberta Filograna[4], Peter Stenvinkel[1,†], Anna Witasp[1,†]

[1] Karolinska Institutet, Department of Clinical Science, Intervention and Technology, Division of Renal Medicine, SE-141 52 Huddinge, Sweden; peter.stenvinkel@ki.se (P.S.); anna.witasp@ki.se (A.W.)
[2] Karolinska Institutet, Department of Clinical Science, Intervention and Technology, Division of Transplantation Surgery, SE-141 52 Huddinge, Sweden
[3] Glasgow Geroscience Group, School of Molecular Biosciences, University of Glasgow, Glasgow G12 8QQ, UK
[4] Karolinska Institutet, Department of Medical Biochemistry and Biophysics, SE-171 65 Solna, Sweden
* Correspondence: angelina.schwarz@ki.se
† These authors contributed equally to this work.

Supplementary materials

Supplemental Table S1 Estimated biological age by skin autofluorescence (SAF) measurements and coronary artery calcium (CAC) score in 211 CKD5 patients according to tertiles of *mtND1*-cn.

***MtND1*-cn-tertiles**	**Low N=69**	**Medium N=70**	**High N=72**	**All N=211**	**N data**	**p-value**
MtND1, cn	51 (34-64)	116 (94-138)	217 (179-279)	119 (65-180)	211	<0.001
Males, n (%)	40 (58)	49 (70)	53 (73.6)	142 (67.3)	211	0.12
Age (years)	50 (32-59)	48.5 (38-59)	41 (30-54)	47 (32-58)	211	0.055
Age by SAF (years)	97 (78-115)	95 (70-111)	82 (65-95)	90 (70-107)	174	0.002
BMI, kg/m²	25 (22.5-27.6)	24.3 (22.1-27.5)	24 (22.3-26.2)	24.2 (22.3-26.9)	209	0.28
Creatinine, µmol/L	644 (522-816)	732 (589-859)	742 (632-942)	720 (581-877)	211	0.033
CT-total	8 (0-508)	11 (0-271)	0 (0-14)	0 (0-126)	159	0.011
Medial calcification score, n (%)					177	<0.001
0	6 (8.7)	13 (18.6)	21 (29.2)	40 (19)		
1	21 (30.4)	26 (37.1)	26 (36.1)	73 (34.6)		
2	12 (17.4)	15 (21.4)	15 (20.8)	42 (20.8)		
3	8 (11.6)	7 (10.0)	7 (9.7)	22 (10.4)		
FRS (%)	6.8 (3.2-16.1)	7.8 (4.3-15.7)	4.8 (2.4-11.7)	6.9 (3.2-14.8)	201	0.16

SAF = skin autofluoresence, BMI = body mass index, CAC = coronary artery calcium, CT total = CAC scores obtained by cardiac computed tomography [11], FRS = Framingham CVD risk score, p-values obtained from Kruskal-Wallis test.

Supplemental Table S2 Estimated biological age by skin autofluorescence (SAF) measurements and coronary artery calcium (CAC) score in 211 CKD5 patients according to tertiles of *mtCOX1*-cn

***MtCOX1*-cn-tertiles**	**Low N=69**	**Medium N=70**	**High N=72**	**All N=211**	**N data**	**p-value**
MtCOX1, cn	34 (25-44)	84(71-100)	156 (131-267)	85 (45-132)	211	<0.001
Males, n (%)	40 (58.0)	51 (72.9)	51 (70.8)	142 (67.3)	211	0.13
Age (years)	49 (32-59)	49 (38-58)	42 (32-56)	47 (32-58)	211	0.18
Age by SAF (years)	95 (78-113)	99 (74-115)	82 (65-95)	90 (70-107)	174	<0.001
BMI, kg/m^2	25.1 (22.4-28)	24 (22.1-27.4)	24.1 (22.5-26.3)	24.2 (22.3-26.9)	209	0.39
Creatinine, µmol/L	661 (555-816)	732 (560-857)	742 (632-942)	720 (581-877)	211	0.05
CT-total	6 (0-508)	9.5 (0-259)	0 (0-26)	0 (0-126)	159	0.043
Medial calcification score, n (%)					177	<0.001
0	7 (10.1)	12 (17.1)	21 (29.2)	40 (19)		
1	22 (31.9)	26 (37.1)	25 (34.7)	73 (34.6)		
2	11 (15.9)	12 (17.1)	19 (26.4)	42 (20.8)		
3	7 (10.1)	11 (15.7)	4 (5.6)	22 (10.4)		
Framingham risk score (%)	6.7 (2.5-15.8)	7.8 (4.2-16.4)	5 (2.7-12.5)	6.9 (3.2-14.8)	201	0.22

SAF = skin autofluoresence, BMI = body mass index, CAC = coronary artery calcium, CT total = CAC scores obtained by cardiac computed tomography [11], FRS = Framingham CVD risk score, p-values obtained from Kruskal-Wallis test.

Supplemental Table S3. Predictors of vascular medial calcification in 177 CKD5 patients

**Variables**	**Coeff**	**95% CI**	**p-value**
Sex, female	-1.52	-2.39 – 0.66	0.001
Age, years	0.08	0.05 – 0.12	0.0001
MtND4 middle tertile vs low	0.13	-0.78 – 1.04	0.78
MtND4 high tertile vs low	0.18	-0.75 – 1.11	0.70

Multivariate logistic regression analysis; pseudo r= 0.21, p=0.0001

Medial calcification scores were grouped into 0+1 and 2+3 groups, respectively

There were missing values for one or more of the parameters in 34 patients

Supplemental Table S4. Clinical characteristics of patients one year after kidney transplantation

	**Total N=32**	**Female N=9**	**Male N=23**	**p-value**
Age, years	49 (33-60)	51 (33-63)	48 (33-59)	0.785
BMI, kg/m^2	25.3 (23.4-26.9)	25.1 (22.4-28.1)	25.6 (23.4-27.6)	0.548
SBP, mmHg	135 (125-144)	139 (125-149)	134 (124-142)	0.389
DBP, mmHg	83 (78-90)	80 (78-91)	83 (73-90)	0.799
P-Creatinine, mmol/L	121 (111-131)	118 (74-134)	121 (113-130)	0.346
eGFR, ml/min	63 (53-75)	54 (45-88)	64 (57-72)	0.785
hsCRP, mg/L	0.8 (0.5-2.7)	1.0 (0.6-4.3)	0.8 (0.5-2.1)	0.498
Cholesterol, mmol/L	4.8 (4.3-5.5)	5.1 (3.7-6.4)	4.8 (4.3-5.4)	1.0
Triglycerides, mmol/L	1.6 (1.2-2.9)	1.3 (0.8-1.5)	1.4 (1.3-1.5)	0.833
HDL-c, mmol/L	1.2 (1-1.2)	1.4 (1.1-¤)	1.2 (1-1.2)	0.466
LDL-c, mmol/L	2.2 (1.6-3)	2 (-)	2 .2(1.5-3.1)	0.510
Albumin, g/L	38 (36-40)	38 (36-41)	38 (36-40)	0.981
Urea, mmol/L	7.4 (6.2-10.3)	8.6 (5.9-10.7)	7.4 (6.2-9.3)	0.66
Sodium, mmol/L	140 (139-142)	140 (138-142)	141 (139-141)	0.464
Potassium, mmol/L	4.0 (3.7-4.3)	3.9 (3.3-4.3)	4.0 (3.7-4.3)	0.37
Hemoglobin, g/L	138 (120-144)	128 (114-142)	139 (126-147)	0.198
Homocystein, μmol/L	19 (14-24)	14 (14-24)	20 (15-24)	0.220

BMI = body mass index, eGFR = estimated glomerular filtration rate (CKD-EPI 2021 formula [51]), SBP = systolic blood pressure, DBP = diastolic blood pressure, hsCRP = high sensitivity C-reactive protein, HDL-c = high density lipoprotein cholesterol, LDL-c = low density lipoprotein cholesterol, ¤ = only one or two sample points were available, p-values obtained from nonparametric Mann-Whitney U test, values are presented as median with IQR

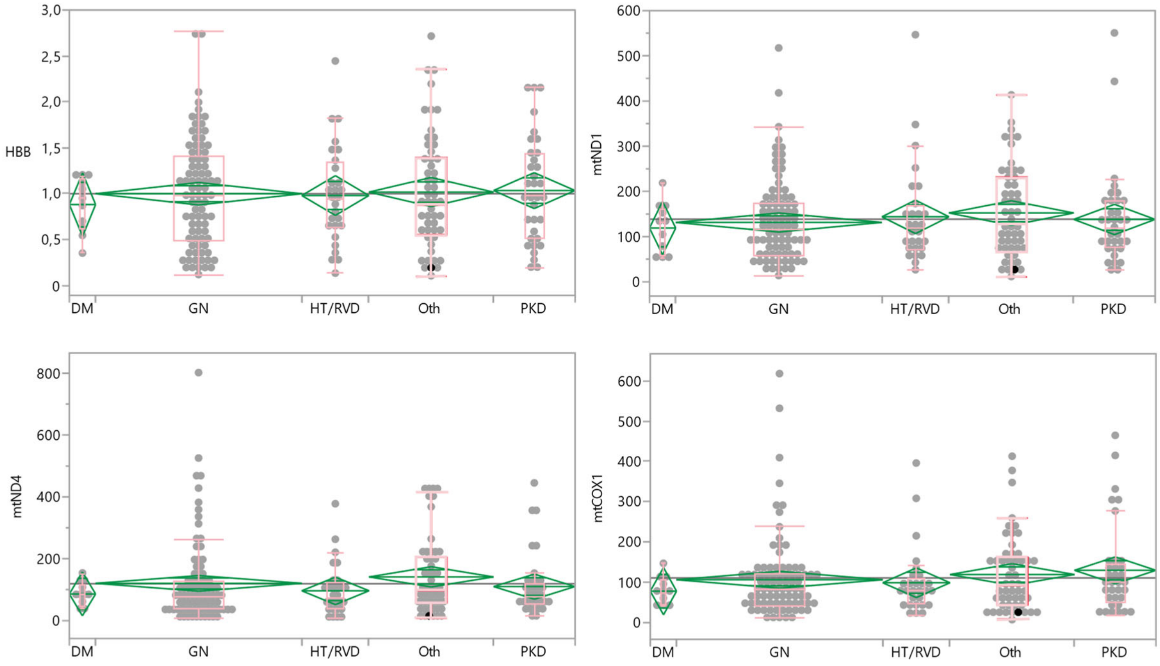

Supplemental Figure S1 MtDNA-cn in patients stratified according to CKD etiology. There is no significant difference in median mtDNA-cn between groups. DM = diabetes mellitus/diabetic nephropathy, GN = glomerulonephritis, HT/RVD = hypertension/renal vascular disease, Oth = other causes or unknown, ADPKD = adult polycystic kidney disease.

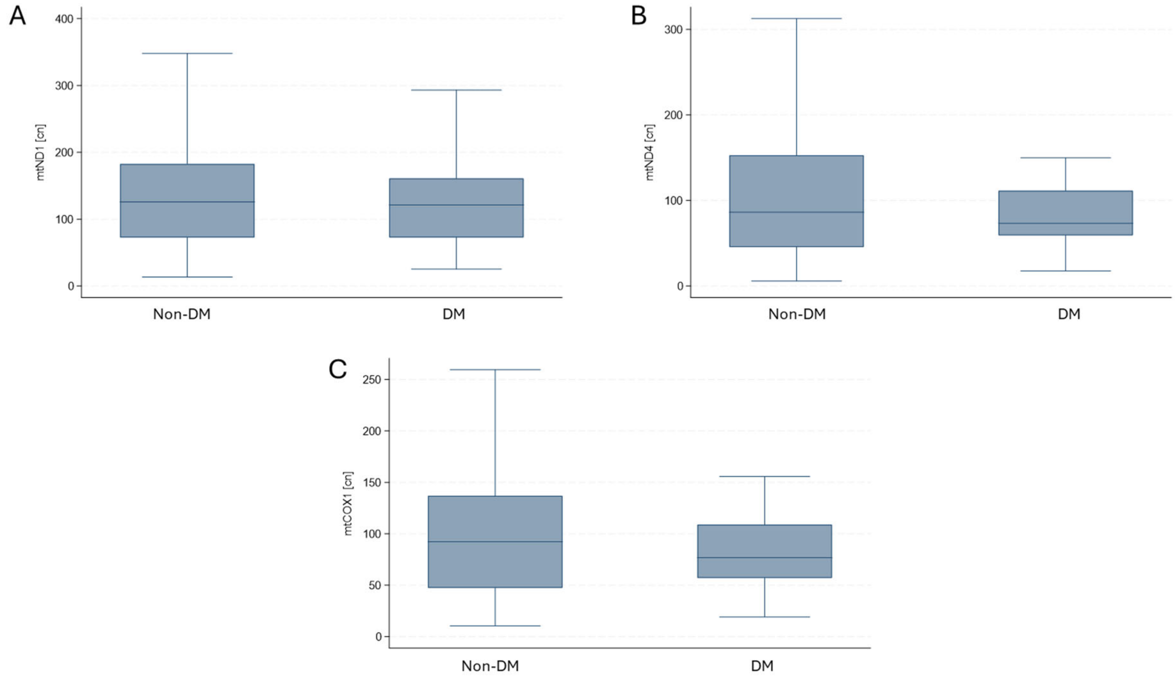

Supplemental Figure S2 MtDNA-cn in CKD5 patients stratified according to presence of diabetes mellitus (DM). There were no significant cn differences between patients with (n=20) or without (n=181; missing n=10) diabetes. (A) *mtND1* 121 *vs* 126, p=0.981; (B) *mtND4* 73 *vs* 86, p=0.359; (C) *mtCOX1* 76 *vs* 92, p=0.166. Groups were compared with Mann Whitney U-test.

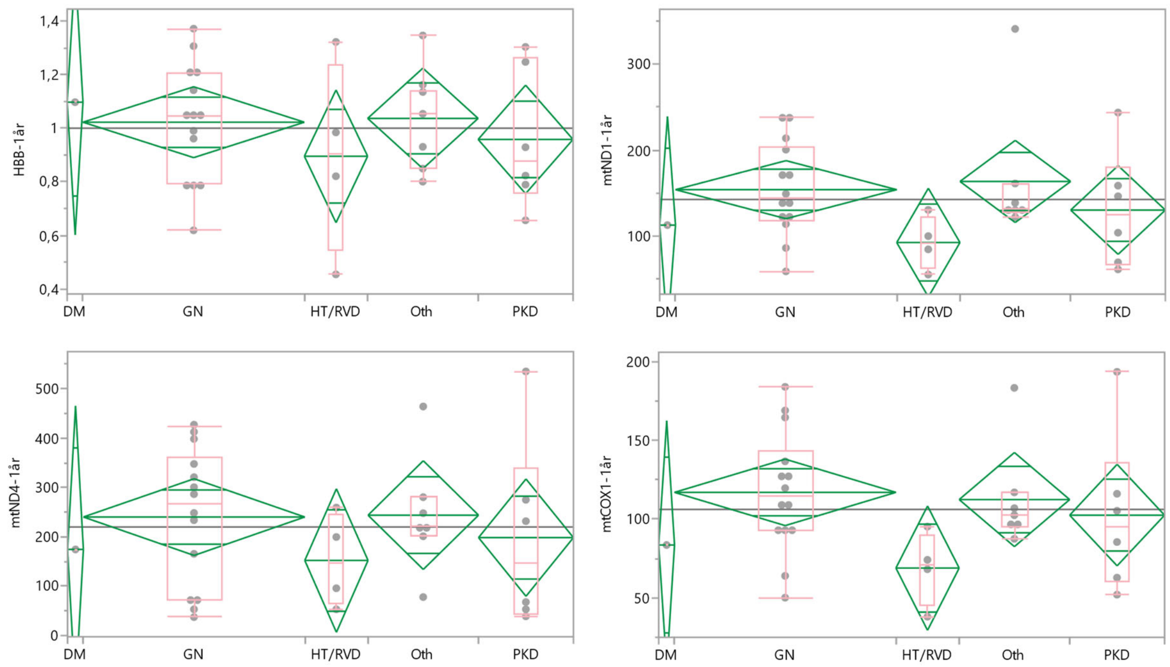

Supplemental Figure S3 MtDNA-cn at one-year-follow-up according to disease etiology. There is no significant difference in median mtDNA-cn at one-year-follow-up, when analyzed according to their original disease etiology before RTx. DM = diabetes mellitus/diabetic nephropathy, GN = all glomerulonephritis, HT/RVD = hypertension/renal vascular disease, Oth = other causes like genetic or unknown, ADPKD = adult polycystic kidney disease, 1år = one year.
